# 0097. Myeloid-derived suppressor cells attenuate inflammation and improve the survival of microbial sepsis

**DOI:** 10.1186/2197-425X-2-S1-P9

**Published:** 2014-09-26

**Authors:** L Tong, GX Hu, JC Cai

**Affiliations:** The 1st Affiliated Hospital, Sun Yat-Sen University, Surgical Intensive Care Unit, Guangzhou, China

## Introduction

Myeloid-derived suppressor cells (MDSCs) are heterogeneous population of immature myeloid cells with suppressive activity, containing precursor of granulocytes, macrophages, and dendritic cells [1–3]. They expand in inflammatory conditions and regulate immune responses. However, the role that MDSCs played in other chronic or acute inflammatory conditions like sepsis is not well understood.

## Objectives

To find the role of MDSCs in sepsis and their relationship with the inflammatory cytokines.

## Methods

The model of caecal ligation and puncture (CLP) was used to induce polymicrobial sepsis in adult male BALB/c mice. Mice were killed under anesthesia, then blood, peritoneal lavage fluid, and organs were harvested. After the spleen MDSCs were isolated, 30 BALB/c mice were randomized divided into three groups which were CLP+MDSCs (day3),CLP+MDSCs (day 10), CLP+saline whereas MDSCs were adoptive transferred into these mice respectively. And the MDSCs accumulation in spleen were observed at day1,3,6,10.

## Results

MDSCs progressively accumulated in spleens during sepsis in survived mice. They were increasing from ~8% at day 1 to ~50% at day 10. Adoptive transfer of late MDSCs into naïve mice after CLP could increase the level of IL-10, decrease the level of INF-γ, and improve the survival rate.

## Conclusions

MDSCs could influence the inflammation response after CLP. Contrasting with the deleterious role of MDSCs in tumor associated diseases, the protective role of these cells was appeared during the early period of polymicrobial sepsis. This may provide a new direction for the treatment of sepsis.
